# Massage and Storytelling Reduce Aggression and Improve Academic Performance in Children Attending Elementary School

**DOI:** 10.1155/2017/5087145

**Published:** 2017-01-19

**Authors:** Lia Lopes Gonçalves, Mariana Callil Voos, Maria Helena Morgani de Almeida, Fátima Aparecida Caromano

**Affiliations:** Department of Physical Therapy, Speech Therapy and Occupational Therapy Rehabilitation Sciences Post Graduation Program, Medical School, University of São Paulo, São Paulo, SP, Brazil

## Abstract

**Background:**

Aggressive behaviors must be addressed in elementary schools. Massage and storytelling can be strategies to deal with aggression because both involve experience exchange and social interaction. Both can decrease stress and anxiety and increase self-esteem.

**Objective:**

To evaluate the effect of two interventions (massage and storytelling) on aggressive behaviors and academic performance of elementary school children.

**Method:**

Three groups (*n* = 35 children in each group) of the second grade participated (aged 6.5–8.1 years). One group received ten extra classes of massage (MG), another group received extra classes of storytelling (SG), and the control group received extra classes of random subjects (CG). Extra classes lasted for 50 minutes, once a week. Aggressive behaviors were recorded on diaries, by the teachers and the coordinator. The frequency of aggressive behaviors and the academic performance of MG, SG, and CG were observed for six months and the groups were compared.

**Findings:**

ANOVAs evidenced that MG and SG, but not CG, showed a reduction in aggressive behaviors registered by the teachers and coordinator, after the intervention. Academic performance of MG and SC improved after the intervention (*p* < 0.05).

## 1. Introduction

Aggressive behavior for elementary school children is expressed as physical or verbal aggression [1–3]. Aggressive behaviors may be related to personal characteristics (e.g., lack of coping strategies, anxiety, depression, low intelligence coefficient, and attention deficits) and/or environmental factors (e.g., school performance pressures and competitions, peer relationship, and family issues) [1, 4, 5]. Aggressive behaviors in elementary schools instigate teacher-student conflicts and peer rejection and represent the primary reason for suspensions and expulsions. Children showing aggressive behaviors are more likely to face difficulties in learning and establishing positive relationships with teachers and classmates [1, 4].

Aggression in children aged up to three years is associated with difficulty communicating their needs to their parents, caregivers, and other children. For older children, between the ages of four and eight, such behaviors are related to difficulty learning appropriate, nonaggressive ways of communicating when faced with a difficult situation [3, 6, 7]. Other factors that may be associated with aggressive behaviors are family difficulties; learning disorders, such as dyslexia; neurological or behavioral disorders, for example, attention deficit/hyperactivity disorder and/or oppositional defiant disorder; emotional trauma, such as domestic violence or sexual abuse; and exposure to violent television shows and movies [8].

Studies about aggression usually focus on the children and their families, and little attention is given to school environment contingencies. Preventive actions against aggressive behaviors must be based on multidisciplinary approaches discussing values, lifestyle, and citizenship. Health promotion in schools is a current concern. An effective school health program can be one of the most cost effective investments a nation can make to simultaneously improve education and health [9].

School health programs aim to improve students' quality of life by promoting education and health and by offering disease prevention and coping with vulnerabilities that may compromise development [9]. Increased attention has been placed on the promotion of students' social and emotional competence to overcome poor academic motivation, dropouts, bullying/aggression, and mental health problems [4–6, 10, 11].

School engagement depends on several factors, such as behavioral engagement (participation in academic and nonacademic activities at school with positive conduct), emotional, and cognitive engagement [6]. Actions can address behavioral engagement in various ways, focusing on participation in academics (homework) and school-related extracurricular activities and motivating positive conduct (e.g., following classroom rules, cooperating with peers during classes) [12, 13].

Children's lack of behavioral engagement is associated with increased academic failure in elementary, middle, and high school (e.g., [13]). Negative developmental pathways of child-reported behavioral engagement have been associated with increased internalizing (e.g., anxiety and depression) and externalizing (e.g., aggression, delinquency, and drug abuse) behaviors [4, 14]. The cause of aggressive behaviors may be due to self-defense, being placed in a stressful situation, lack of routine, extreme frustration or anger, inadequate speech development, overstimulation or exhaustion, lack of adult supervision, and mirroring the aggressive behaviors of other children [1, 2, 15].

Massage can be practiced at school as a strategy to minimize aggressive behaviors [16, 17]. Touching and being touched are key actions in human development that increase body awareness, relax muscles, and promote physical, motor, neurological, and intellectual development. Touch enables the generation of mental images, emotions, and feelings and produces metabolic and physiological changes, especially in the immune, endocrine, neuromuscular, and cardiac systems [18, 19].

Currently, research about the benefits of massage in children focuses on attention, learning, immune function, bonding, self-esteem, body perception, and social/emotional/motor behavior [16–19]. The findings support massage as a resource of physical, emotional, and social health promotion. However, these studies are methodologically limited and rarely carried out at schools [17, 19].

Another strategy that can improve social behavior is storytelling. It encourages written and oral communication, promotes better bonding between teachers and children, and facilitates the comprehension, control, and expression of thoughts and feelings. Storytelling can be a recreational, educational, instructional, and emotional strategy to work with children. It broadens the social, cultural, and intellectual repertoires and helps to reduce stress and anxiety [20]. Storytelling promotes joy and confidence, as it instills virtues in children and makes them aware of their culture and other cultures. It enhances verbal proficiency and improves attention, concentration, creativity, imagination, and memory [21].

Both massage and storytelling promote confidence, relaxation, well-being, empowerment, and socialization. Both can improve attention and concentration and optimize respect, self-caring, affection, and perception. Based on these facts, this study aimed to evaluate the effect of massage and storytelling in aggressive behaviors at school and academic performance of elementary school children.

## 2. Method

### 2.1. Participants

This study was approved by the Research Ethics Committee of the Medical School of University of São Paulo (process number 11369). Students from the second grade of a public elementary school of São Paulo (*n* = 105), aged 6.5 to 8.1 years (mean age 7.4, SD 0.4), 56 boys and 49 girls, participated. Fifty-two were declared as mulatto, 22 as black, and 31 as white, according to the enrollment file, filled by parents. Three classes of students were attending the second year of elementary school in a public school in the city of São Paulo. The classrooms were randomly assigned to have 10 classes of massage (MG), storytelling (SG), or a random extra class of a curricular discipline (CG). All parents were informed about the study and gave informed consent prior to participation.

The school enrolled children between 3 and 14 years old. School time lasted for four hours and a half per day (Monday to Friday). Four hours were spent with regular classes and they had a 30-minute break. They practiced sports in the physical education classes, three times a week for 45 minutes. Experts in arts and music also gave 45 minutes classes in the regular curriculum, once a week each class. Children belonged to low-income families and lived near the school. Students received daily snacks in break time and powdered milk to take home monthly, school supplies, and uniforms. Only children who attended at least 85% of classes received the powdered milk to take home, which is part of a Brazilian program to reduce school evasion. In the present study, no children evaded school during data collection.

A typical school year in Brazil includes classes from March to June, one month winter holidays in July, and classes from August to November. Children may also have one or two weeks in February and December, but mostly for adaptation (February) and finalization (December) activities.

### 2.2. Massage Group

The teacher of MG was trained by the researcher for 4 hours, prior to the beginning of the massage classes. During the massage classes, children were randomly assigned in pairs. Each child sat facing his or her assigned partner, and massage techniques were demonstrated by the teacher. Children wore cotton T-shirts and performed the massage maneuvers on each other, on shoulders, arms, hands, and scalp. Classes lasted for 50 minutes, weekly, always in the same day of the week. After the first class, every day, before the snack break, children were asked to practice the massage with randomly determined pairs, for 10 minutes, being 5 minutes to give and 5 to receive massage.

### 2.3. Storytelling Group

The teacher of SG was trained by the researcher for 4 hours, before giving the storytelling classes. A list of fifteen books was selected, based on the age of participating children. The books approached the themes friendship, family, reflecting about relationships, expressing feelings, respecting differences, working cooperatively, imagination and creativity, health, empathy, environment, culture, science, philosophy, history, and geography.

The weekly storytelling classes lasted for 50 minutes and were always scheduled on the same day of the week. Every week, students chose one book from three offered by the teacher. Children sat in circle, on cushions, and the teacher told the story. Children could ask questions and make comments during the class. Every day, before the snack break, children were asked to retell the story of the week to a randomly determined pair, for 10 minutes, being 5 minutes to listen and 5 to tell the story.

### 2.4. Control Group

Students chose one of the regular disciplines (Portuguese, History, Geography, Mathematics, Physical and Biological Sciences, Physical Education, Arts, and Music) to have a 50-minute extra class. A teacher gave the class and children were free to ask questions and comments. The extra class was given weekly, always in the same day of the week. After the first class, every day, before the snack break, children were asked to recall the subject of the week with a randomly determined pair, for 10 minutes, being 5 minutes to listen and 5 to explain the subject.

### 2.5. Aggressive Behaviors Registering

Prior to the interventions, the teachers of the three participating groups and the coordinator were trained for eight hours by another researcher (not the same who gave instructions about massage and storytelling classes) to register on a diary the episodes of verbal or physical aggression, based on the observation of students and on parents' complaints. The records were performed for four months: two months before and two months after the interventions. The teachers were instructed to write one or two paragraphs reporting each observed incident. They had twenty minutes to perform this task daily. The teachers were not informed about the hypothesis/aims of the present study.

After that, an examiner, blind to the teachers, groups, interventions, and objectives of the study, read the diaries and filled the chart of aggressive behaviors, with tic marcs. Examples of aggressive behaviors were provided for this examiner as follows.

*Examples of Aggressive Behaviors, Given to the Teachers Prior to Data Collection*

*Verbal Aggression*

  Gossiping
  Mimicking
  Name calling
  Posting rumors/ embarrassing information
  Sending hurtful e-mails
  Teasing
  Threatening
  Yelling

*Physical Aggression*

  Biting
  Hitting
  Kicking
  Pulling one's hair
  Punching
  Pushing
  Spitting

*Other Types of Aggression*

  Breaking one's objects
  Posting embarrassing pictures or videos
  Taking one's objects

### 2.6. Academic Performance

Considering all curricular disciplines given (Portuguese, History, Geography, Mathematics, Physical and Biological Sciences, Physical Education, Arts, and Music), a mean grade was calculated for each child and then, for each group. The performance on disciplines was evaluated by exams in the last week of April (before intervention) and September (after intervention). The means of the three groups were compared before and after the intervention.

### 2.7. Data Analysis

**Figure 1:**
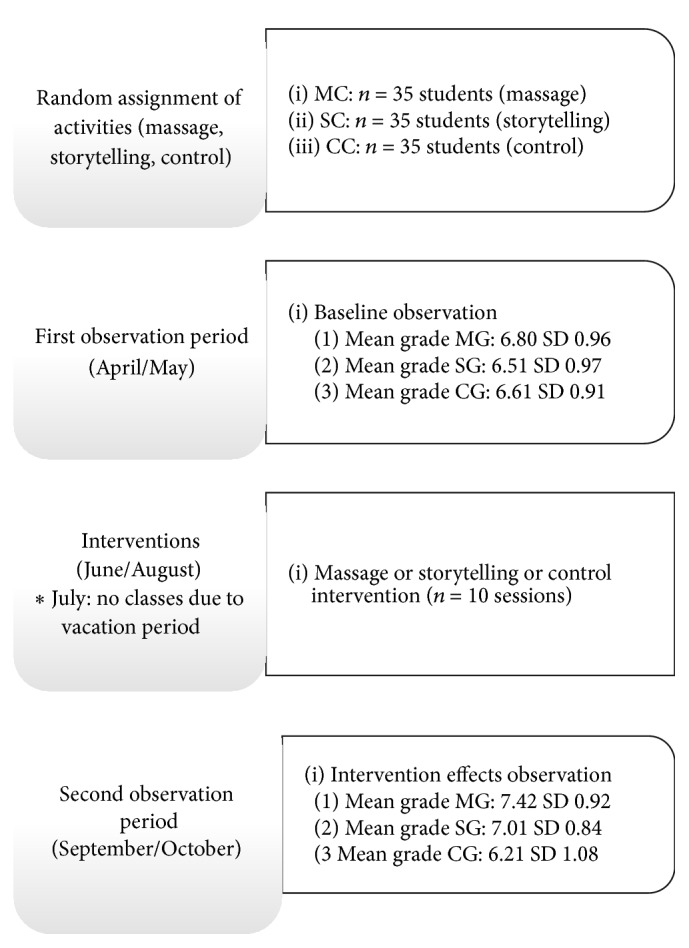
Research protocol.

The numbers of registers of aggressive behaviors made by the teachers and the coordinator and the mean grades of the groups showed normal distributions and were compared by analyses of variance (ANOVA for repeated measures and post hoc Tukey tests). Alfa was set as < 0.05. We selected the periods of observation April/May and September/October and the period of intervention June/August. We believed that children would be less influenced by the beginning and the end of the school year in these periods. In the beginning of school year (February/March), children may need some time to know each other and to adapt to the routine and group. In the end, they may be worried about the final exams (November/December). The research protocol is represented in Figure 1.

## 3. Results

### 3.1. Teachers and Coordinator's Observations

**Figure 2:**
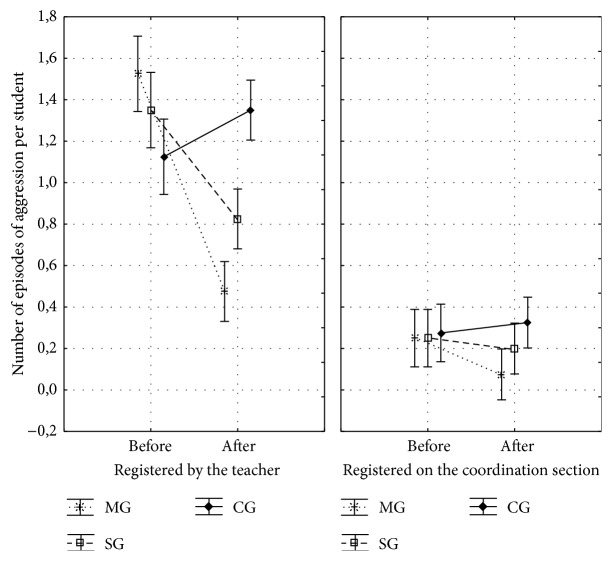
Records of teachers and coordination register books, in which aggression episodes involving students and students and parents complaints are registered. Before the intervention: observations from April/May were analyzed. After the intervention: observations from September/October were analyzed.

Analyses of variance compared the number of episodes of aggressive behaviors registered by the teachers and the coordinator on their diaries during the period before and after the interventions. We found a significant interaction between groups and periods, with F2,117 = 23.49; *p* < 0.001; effect size = 0.287. The post hoc Tukey test showed that the number of episodes of aggressive behaviors was significantly lower in MG (*p* < 0.001) and SG (*p* = 0.002) compared to the CG after the interventions (Figure 2).

Both teacher and coordination's records showed a reduction in the number of aggressive behaviors after the intervention and the teacher's records reached statistical significance (MG: 1.5 registers per student before the intervention to 0.5 registers per student after the intervention; SG: 1.4 registers per student before the intervention to 0.8 registers per student after the intervention).

### 3.2. Academic Performance

**Figure 3:**
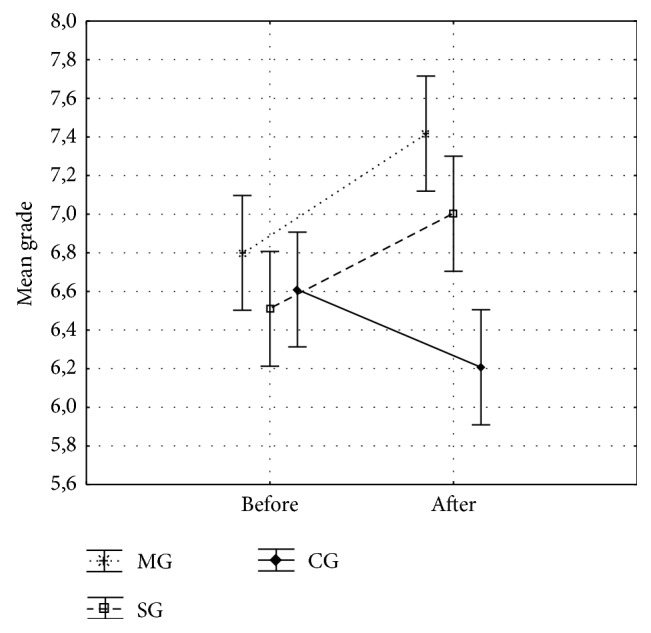
Mean grade obtained by each group before and after the intervention. Before the intervention: the exams of the last week of April were considered. After the intervention: the exams of the last week of September were considered.

Analyses of variance compared the mean grades of each group during the period before and after the interventions. We found a significant interaction between groups and periods, with F2,117 = 10.19; *p* < 0.001; *η* = 0.149. The post hoc Tukey test showed that the mean grade was significantly higher in the MG (*p* = 0.038) and SG (*p* = 0.045) compared to the CG after the interventions (Figure 3).

The mean grade of MG was 6.8 before the intervention and 7.4 after the intervention. This group had the highest mean grade in the period studied, exceeding the expectation of school. The mean grade of the SG increased from 6.5, before the intervention, to 7.0, after the intervention.

## 4. Discussion

This study showed that classes of massage and storytelling reduced the number of aggressive behaviors and improved academic performance in 6–8-year-old-students attending elementary school. After the intervention period, we found a reduction in the number of types of verbal/physical aggression, meetings with the coordination due to inappropriate behavior, and parental complaints in the MG and SG groups.

The interventions of massage and storytelling provided benefits for children, as observed in the study by Powell and Potter [16], who showed that fifteen meetings in six months, with relaxation, yoga, and interactive games, reduced helplessness and aggression. Aggressive behaviors can be addressed in 6–8-year-old children by interventions that emphasize a positive model, of respect and affection [4]. Conversely, punishments and authoritarian relationships between children, teachers, and caregivers may not have such beneficial effects [6]. Besides, activities based on adults' expectancies may lead to aggression, as a form of resistance to frustration and academic failure.

Diversified educational interventions have implemented teaching and learning and have showed beneficial results. Approaches that promote the reflection and the understanding about new contents and experiences usually facilitate teaching and learning processes. Teachers can promote a higher interaction with and between the students. When students adopt a more active and protagonist posture, they can also detect and verbalize their needs and difficulties, as well as their possibilities and availabilities. Relationships at school can become a positive model of interaction and modify other relationships. Students can become more confident to express how they feel and to show more empathic and helpful behaviors.

### 4.1. The Beneficial Effects of Massage

Massage reduced the number of aggressive behaviors and improved academic performance in 6–8-year-old students attending elementary school. Massage affects the interaction with colleagues, extending friends networks. It increases the confidence to deal with vulnerabilities [16, 17, 19]. Touching and being touched involve acceptance and trust, exchange of physical experiences, and awareness of others. Opposite, cultures with rules and prohibitions may affect people's physical contact behavior and its benefits. Massage allows the experience of physically interacting with another person in the situation of being cared. Children experienced the situation of being the caregiver and providing something positive for someone else [17, 19].

Children aged 6–8 years are transitioning to the concrete operational stage, according to Piaget's theory of cognitive development [22]. This stage is characterized by the elimination of egocentrism, which is the inability to understand a perspective other than one's own. During this stage, the child acquires the ability to view things from another individual's perspective, even if they think that perspective is incorrect, which is practiced in massage classes. Therefore, massage contributes for this development stage.

### 4.2. The Beneficial Effects of Storytelling

Storytelling reduced the number of aggressive behaviors and improved academic performance in 6–8-year-old students attending elementary school. Children of SG showed improvement in social behavior. Our findings are in agreement with the ones reported in the literature, showing that storytelling decreases anxiety and provides joy, confidence, and relaxation [10, 20]. Storytelling stimulates creativity, language, and memory, promoting the healthy development and coping processes in situations of social disorganization or illness [21].

Children aged 6–8 years are in the transition to the concrete operational stage, according to Piaget's theory of cognitive development [22]. This stage is characterized by the appropriate use of logic, with thoughts more mature and “adult like,” which can be extensively practiced in storytelling. Telling and retelling stories provide children projection and introspection, inspired by the struggles of the characters, fighting their battles and enjoying the pleasure of defeating giants and monsters. These projections can improve empathic and cooperative behaviors and reduce aggression.

To sum up, both massage and storytelling seemed to facilitate interpersonal connection. Both approaches seemed to improve not only self-caring and self-regulation, but also affection, empathy, and respect. Such changes impacted positively in social behavior and academic performance at school.

### 4.3. Other Impacts on the Community

We found teachers who were motivated and available to continue with the massage and storytelling classes. The activities were offered to all students aged 5–9 years as extra classes, after the postintervention observation period.

Teachers of Physical Education and Portuguese became engaged in organizing massage and storytelling activities, respectively. We believe that this action is the result of the interest of teachers in children's mental health, which contributes to the humanization of educational processes. Massage and storytelling can act in line with educational goals, as a way of approaching collaborative and motivating new educational projects.

Children who exhibit high rates of conduct problems at school often experience chronic difficulties in both academic and social domains. Many of these students come from a background of early adversity (poverty, family instability, parenting difficulties, and violence exposure) [1, 6, 23]. The high rate of school failure and dropout experienced by students with conduct problems, compared with other students with disabilities, underscores the difficulties schools face in serving these children effectively [6]. For this reason, strategies with positive effects on aggressive behaviors are important to assure better social and academic development of elementary school children and teachers.

### 4.4. Limitations

Although teachers were not informed about the hypothesis and aims of the present study, they may have made some inferences correlating massage, storytelling, and aggressive behaviors. We had to keep the same teachers giving the extra class and assessing aggressive behaviors of each group (one teacher for MG, another for SG, and another for CG), due to professional/time unavailability. Another limitation is that we could not randomly assign the students to the groups. Also, evaluating only one (public) school is a limitation, because children living in different social contexts were not compared.

### 4.5. Future Studies

Future studies should assess larger samples and interview parents about possible beneficial effects of massage and storytelling outside school environment.

## 5. Conclusion

Interventions with massage and storytelling seemed to affect behavior and academic performance positively in 6–8-year-old children from an elementary public school of São Paulo, Brazil. These strategies should be explored as children's education and health promotion approaches.

## Additional Points

*Relevance to Clinical Practice*. Massage and storytelling positively seemed to affect the behavior and academic performance of elementary school children.* Limitations*. Although we followed 105 children, only one public school participated in the study.* Recommendations for Future Research*. To investigate the impacts of massage and/or storytelling in longer follow-ups and other age ranges.
